# Psychosocial Impacts of the COVID-19 Quarantine: A Study of Gender Differences in 59 Countries

**DOI:** 10.3390/medicina57080789

**Published:** 2021-07-31

**Authors:** Stephanie A. Kolakowsky-Hayner, Yelena Goldin, Kristine Kingsley, Elisabet Alzueta, Juan Carlos Arango-Lasprilla, Paul B. Perrin, Fiona C. Baker, Daniela Ramos-Usuga, Fofi Constantinidou

**Affiliations:** 1 Magellan Federal, Silver Springs, MD 20910, USA; 2 JFK Johnson Rehabilitation Institute, Edison, NJ 08820, USA; yelena.goldin@hackensackmeridian.org; 3 Institute of Cognitive and Emotional Wellness, Westchester, NY 10801, USA; cognitiveandemotionalwellness@gmail.com; 4 Center for Health Sciences, SRI International, Menlo Park, CA 94025, USA; elisabetalzueta@gmail.com (E.A.); fiona.baker@sri.com (F.C.B.); 5 Department of Biological and Health Psychology, Autonomous University of Madrid, 28049 Madrid, Spain; 6 Biocruces Bizkaia Health Research Institute, 48903 Barakaldo, Spain; jcalasprilla@gmail.com (J.C.A.-L.); daniela.ramos.usuga88@gmail.com (D.R.-U.); 7 Department of Cell Biology and Histology, University of the Basque Country, 48015 Leioa, Spain; 8 IKERBASQUE, Basque Foundation for Science, 48009 Bilbao, Spain; 9 Department of Psychology, Virginia Commonwealth University, Richmond, VA 23284, USA; pperrin@vcu.edu; 10 School of Physiology, University of the Witwatersrand, Johannesburg 2131, South Africa; 11 Biomedical Research Doctorate Program, University of the Basque Country (UPV/EHU), 48940 Leioa, Spain; 12 Center for Applied Neuroscience, Department of Psychology, University of Cyprus, Nicosia 1678, Cyprus; fofic@ucy.ac.cy

**Keywords:** trauma-related distress, stress, depression, anxiety, sleep quality, exposure, coping, gender, coronavirus disease 2019 (COVID-19)

## Abstract

*Background and Objectives*: There is strong evidence in the literature that women experience psychological disorders at significantly higher rates than men. The higher rates of psychological disorders in women may partly be attributable to gender differences in response to stressors and coping styles. The objective of this study was to contribute to the growing body of literature investigating gender differences in mental health outcomes and coping styles during the coronavirus disease 2019 pandemic in a large sample of individuals from 59 countries with variable demographic and socio-cultural characteristics. *Materials and Methods*: Survey data were collected from the general population following a snowball sampling method, and the survey was promoted through social media platforms and mailing lists. Participants included 6882 individuals from the general population from 59 countries around the world. A combination of both standardized and adapted measures was used to create a survey, originally in English and then translated to Spanish, Italian, French, German, and Turkish. *Results*: Compared with men, women presented with higher levels of trauma-related distress; had a harder time decompressing; were more depressed, anxious and stressed; showed decreased frustration tolerance and reported lower quality of sleep and an increased likelihood of taking sleep medication or other natural sleep remedies. Overall, women tended to be more vulnerable during the pandemic in developing symptoms consistent with various forms of mental disorders such as depression, anxiety and post-traumatic distress. However, they also were more likely than men to use a variety of adaptive coping strategies, including concentrating on doing something about the situation and getting emotional support from others. *Conclusions*: A high prevalence of mood symptoms was noted among women. In addition to meeting the physical health needs of the population, emphasis needs to be given to mental health and the prevention of psychiatric disorders, particularly in women.

## 1. Background

### 1.1. COVID-19

The coronavirus disease 2019 (COVID-19) has had a devastating effect on the world. Classified by the World Health Organization (WHO) as a pandemic [1], COVID-19 has been found to cause gastrointestinal and respiratory tract symptoms similar to severe acute respiratory syndrome (SARS) and Middle East respiratory syndrome (MERS) [2]. To date, COVID-19 has been diagnosed in 112,437,714 individuals in 219 countries and territories around the globe [3]. Of these, there have been 2,489,735 deaths and 87,994,483 recoveries. While men and women seem to contract COVID-19 at similar rates, men have higher morbidity and mortality [4,5,6], and more extensive lung disease overall [7]. However, women are more likely to experience psychological disorders and be subjected to intimate partner violence as a result of the COVID-19 quarantine [8,9].

In the absence of pharmaceutical interventions, sheltering in place at home and social distancing were globally deemed as the best strategy to stop the spread of the virus. Although effective in containing the spread of COVID-19, isolation and social distancing caused an interruption in the normal routine of many people in the world, with schools being closed and parents trying to juggle telework, childcare and house management [10]. This has led to changes and disruption of individuals’ mental wellbeing and sleep schedules [11], similar to those observed following previous natural disasters [12,13,14]. To date, only a few studies have examined the changes in sleep quality and mood during the COVID-19 pandemic in both the general population and in healthcare professionals. Casagrande et al. [15] found that 57.1% of responders to an online survey reported poor quality of sleep, 32.1% reported increased symptoms of anxiety, 41.8% reported increased distress and 7.6% reported symptoms of post-traumatic stress disorder (PTSD). Sleep disorders and anxiety disorders were more prevalent in women, those unemployed and those who were worried about being infected with COVID-19 (or who lost loved ones to COVID-19). These findings are consistent with other studies conducted around the world [16,17].

### 1.2. Gender Differences in Psychosocial Functioning

There is strong evidence in the literature that women experience psychological disorders at significantly higher rates than men [18,19,20]. Depression, the most widely studied psychiatric condition, is more prevalent in women [21]. Higher rates of depression in women have been attributed, in part, to biological factors, including genetic factors and stress responsivity [22], as well as environmental factors, including feminization of poverty [23] and higher rates of victimization in women [24,25].

### 1.3. Gender Differences in Exposure and Coping

The higher rates of psychological disorders in women may partly be attributable to gender differences in response to stressors and coping styles [22,26,27]. Two primary coping styles identified in the literature are problem-focused coping and emotion-focused coping. Research has demonstrated that women are more likely to use emotion-focused coping strategies in response to most stressors, while men tend to rely more on problem-focused coping strategies [22,27,28,29]. Although one study found that emotion-focused coping strategies are effective for protecting against depression in women but not men [29], the literature generally supports that problem-focused coping is more effective than emotion-focused coping, since the latter has been associated with greater psychological and functional impairment [30,31]. These gender differences in coping styles have been proposed as significant contributors to the higher rates of depression and anxiety disorders among women in the general population [29]. 

Regarding the COVID-19 pandemic, numerous studies have evaluated the coping strategies used by people during social-isolation periods, e.g., [32,33,34,35], and all of them agreed that a maladaptive coping style was related to higher levels of psychological distress. However, few studies have explored the differences between men and women in coping styles during the pandemic. In the study by Park and colleagues [36], the presence of stress and the coping strategies of 1015 Americans were evaluated through an online survey. Women were more stressed and were more likely to use emotion-focused strategies such as distraction, and emotional and religious support than men. Glenister and colleagues [37] evaluated the psychological impact of the pandemic in a group of 339 rural Australian women living with and without children, and concluded that women living with children were more vulnerable to stress due to poorer health behaviors (e.g., alcohol use) and maladaptive coping strategies. Moreover, when evaluating the relationship between coping strategies and life satisfaction during quarantine in a group of 337 Chinese individuals, Li and colleagues [38] found that positive coping was related to life satisfaction in men but not in women. Interestingly, women used positive coping (emotional and social support-seeking) more than men; however, it was not as effective or adaptive in improving life satisfaction. Finally, Volk and colleagues [39] analyzed coping styles in the face of the COVID-19 pandemic in a sample of 516 Americans, taking personality into account through the HEXACO model [40], as well as sociodemographic factors. The results suggested that the differences in coping styles between men and women were mediated by personality traits so that the use of a positive or negative strategy depended more on the person’s personality than on their gender.

Even though there is extensive literature showing gender differences in mental health, there are few large multi-country studies to date examining the differential impact of COVID-19-related mitigation responses, such as social isolation, between genders. Many of these studies have methodological limitations such small sample sizes [41,42], a focus on a single country [15,16,17,43,44,45,46] or being from a specific region of the world with distinct socio-cultural characteristics [13,47]. The goal of the present exploratory study, therefore, was to contribute to the growing body of literature investigating gender differences in mental health outcomes and coping styles during the COVID-19 pandemic in a large sample of individuals from 59 countries with variable demographic and socio-cultural characteristics. We hypothesized that women would exhibit more symptoms of trauma-related distress, stress, depression and anxiety. Further, we thought women would exhibit poorer sleep quality and coping skills than men.

## 2. Methods

### 2.1. Participants

The participants included 6882 individuals from the general population from 59 countries around the world. Following the World Bank classification system by geographical word region, at the time of data collection, 59.72% of the respondents were living in Latin America and the Caribbean, 24.15% in Europe and Central Asia, 13.91% in North America, 1.21% in Sub-Saharan Africa, 0.96% in East Asia and the Pacific, and 0.06% in South Asia. The primary languages spoken included Spanish (68.1%), English (18.6%), French (3.9%), Italian (3.6%), German (3.3%) and Turkish (2.5%). The participants were mostly women (78.8%), who were married or in a domestic partnership (51.1%), employed/engaged (80.1%), and who were living with more than 1 person (88.3%), with an average age of 42.3 years (*SD* = 13.9) (see Table 1). Given that it is estimated that there are over 1 million individuals in the US alone who are transgender [48], the subsample of 17 transgender or gender non-binary participants, or 0.2% of the total sample, was not considered representative and was removed from all analyses. Only 6.9% of participants rated their pre-COVID-19 health status as fair or poor, while 15.3% reported their current health status as fair or poor. The majority of participants had no history of any chronic health condition such as diabetes, arthritis, asthma, cancer, etc. (73.6%); mental health condition such as depression, schizophrenia, autism, etc. (78.6%); or neurological condition such as epilepsy, traumatic brain injury, multiple sclerosis, etc. (96.2%). 

With regard to household makeup, 11.2% of participants lived with children 5 years of age or younger, 24% lived with children aged 6–17 years and 22.5% lived with children aged over 18 years. The majority (52.8%) lived with a spouse or partner, while a small proportion lived with parents (23.2%), grandparents (2.9%), other family members such as aunts and cousins (11.7%) or other people such as roommates, friends, or tenants (5.8%). The majority of participants were following severe (68.3%) and moderate (28.0%) social distancing restrictions, with 77.0% having left the house only 3 times or fewer during the week prior to the survey; of these, 21.1% had not left the house at all. Further, the majority reported their lives as being much worse (13.7%) or slightly worse (39.9) since the start of the pandemic. 

### 2.2. Procedure

A combination of both standardized and adapted measures was used to create a survey, originally in English and then translated to Spanish, Italian, French, German, and Turkish. The online survey was hosted and distributed using the online platform SurveyMonkey from 19 April to 3 May 2020—when the population of most of the world’s countries were under lockdown measures due to the COVID-19 pandemic. Data were collected following a snowball sampling method, and the survey was promoted through social media platforms (Instagram, Facebook, Twitter and WhatsApp) and mailing lists. Additionally, Facebook ad tools were used to advertise the survey, improving access to a broader range of ages in the general population. The average response time was 15 min.

## 3. Measures

### 3.1. Epidemic–Pandemic Impacts Inventory (EPII)

The EPII is a measure of the personal and social impacts of epidemics and pandemics [49] designed by a team of professionals with expertise in stress, trauma, resilience and coping. This survey utilized the eight questions of the Infection History section of the questionnaire. For example, individuals were asked to check yes (me), yes (person in home) or no to each question regarding “whether the pandemic has impacted you or a person in your home.” Questions included items like: “Currently have symptoms of this disease but have not been tested,” “Tested and currently have this disease,” and “Someone died of this disease while in our home.” Psychometrics and scoring rubrics were not available as this was a newly created measure by a multidisciplinary team at the University of Connecticut. Therefore, this scale was utilized to qualitatively describe the exposure of the sample.

### 3.2. Children’s Revised Impact of Events Scale-8 (CRIES-8)

The CRIES-8 is an eight-item (α = 0.88) measure of trauma-related distress. Half the items measure intrusion and half measure avoidance. Items are scored on a four-point scale including not at all = 0, rarely = 1, sometimes = 3 and often = 4. It has good face and construct validity, and a stable factor structure, and correlates well with other indices of distress [50,51,52,53,54,55,56]. There are two subscales, each with four items: intrusion and avoidance. The CRIES-8 has been shown to have good reliability and validity in samples of children, adolescents and adults aged 8 to 75 years [57,58,59]. It has been translated into more than 20 languages and maintained its factor structure and validity after translation via associations with other indices of trauma and distress [53,60].

### 3.3. Depression, Anxiety and Stress Scale-21 (DASS-21)

The DASS-21 is a short-form, 21-item measure of depression, anxiety and stress. Each self-reported question is rated on a four-point Likert scale including 0 = did not apply to me at all, 1 = applied to me to some degree or some of the time, 2 = applied to me to a considerable degree or a good part of time, and 3 = applied to me very much or most of the time. For the purposes of this study, only the depression (α = 0.91) and stress (α = 0.91) subscales were used. The depression scale is a measure of hopelessness, low self-esteem and low positive affect [61]. The stress scale is a measure of tension, agitation and negative affect. The DASS-21 has been shown to have decent to excellent internal consistency, convergent validity, discriminative validity and diagnostic utility [61,62,63,64].

### 3.4. Generalized Anxiety Disorder-7 (GAD-7)

The GAD-7 [65] is a seven-item measure (α = 0.92) of anxiety and worry rated on a four-point Likert scale with total scores ranging from 0 to 21, with higher scores indicating more anxiety. The GAD-7 has good psychometrics such as strong reliability, construct validity [66,67], internal consistency and convergent validity [68].

### 3.5. Regulatory Satisfaction Alertness Timing Efficiency Duration (RU-SATED)

The RU-SATED scale [69] is a measure of sleep health (α = 0.63) including (1) regularity, (2) subjective satisfaction, (3) alertness during waking hours, (4) appropriate timing, (5) high efficiency and (6) adequate duration. The RU-SATED scale is rated on a three-point Likert scale including 0 = never, 1 = sometimes and 2 = always, with total scores ranging from 0 to 12 and higher scores indicating better sleep health. The RU-SATED scale has been shown to have adequate internal consistency and good item correlation, reliability, and convergent validity [70,71]. Independently of the RU-SATED scale, participants were also asked to report the total number of hours of sleep they had in a 24 h period, and whether they used prescribed or over-the-counter medicine to help them sleep, or natural sleep aids or therapies such as relaxation techniques and herbal supplements. 

### 3.6. Brief Coping Orientation to Problems Experienced (Brief COPE)

The Brief COPE is a 28-item measure (α = 0.72) pared down from the 60-item version designed to measure coping on a four-point Likert scale of 1 = “I haven’t been doing this at all,” 2 = “I’ve been doing this a little bit,” 3 = “I’ve been doing this a medium amount,” and 4 = “I’ve been doing this a lot”. There are 14 subscales with two items each, including active coping, planning, positive reframing, acceptance, humor, religion, using emotional support, using instrumental support, self-distraction, denial, venting, substance use, behavioral disengagement and self-blame [72]. The Brief COPE has been shown to have good internal reliability for the scales [72]. For the sake of brevity, only the first item from each scale was used in the survey, for a total of 14 items. 

## 4. Data Analysis

Descriptive statistics were used to characterize the participants. Means ± SDs were calculated for continuous variables. Percentages were obtained for categorical variables. Statistical analyses were conducted to examine differences between women and men on specific variables. For categorical variables, a series of chi-squares was calculated. A series of one-way ANOVAs was also used to determine between-group differences in six main areas of interest including trauma-related distress, sleep quality, stress, depression, anxiety and coping. Given the number of planned analyses, a conservative alpha level of *p* < 0.01 was set, minimizing the chance of a Type I error. 

## 5. Results

### 5.1. Psychosocial Implications of COVID-19 Pandemic-Related Isolation

#### 5.1.1. Trauma-Related Distress

With regard to trauma-related distress symptoms, the entire sample rated their impact of the COVID-19 pandemic on the CRIES-8. More than half of the participants (71.7%) reported they sometimes or often thought about the pandemic even when they did not mean to. Similarly, the majority of participants endorsed sometimes and often regarding trying to remove the COVID-19 pandemic from their memory (55.7%) and having had strong feelings about the COVID-19 pandemic (53.9%). Most participants denied staying away from reminders about the pandemic (54.3%) and having pictures of it pop into their mind (57.6%). Most participants endorsed having other things continuing to make them think about the pandemic (57.0%) and trying not to think about it (53.5%).

Women had significantly higher intrusion (male: mean = 9.2, SD = 3.3; female: mean = 10.4, SD = 3.2; *F*(1, 6863) = 152.3, *p* < 0.001), avoidance (male: mean = 8.4, SD = 3.5; female: (9.7 (3.4); *F*(1, 6863) = 186.8, *p* < 0.001) and total (male: mean = 17.6, SD = 6.1; female: mean = 20.1, SD = 6.0; *F*(1, 6863) = 210.1, *p* < 0.001) scores on the CRIES-8, indicating significantly more symptom severity regarding trauma-related distress than men.

#### 5.1.2. Stress

The stress subscale of the DASS-21 revealed significant differences between women and men (*F*(1, 6863) = 134.3, *p* < 0.001) with regard to stress during the COVID-19 pandemic. Women, on average (mean = 15.2, SD = 10.7), scored higher, indicating more stress symptoms, than men (mean = 11.6, SD = 10.0) (see Table 2). Overall, the scores indicated that men were endorsing normal stress symptoms, while women exhibited mild stress-related symptoms. Women were more likely to endorse having trouble winding down (38.5% vs. 26.9%; *X*^2^ (3) = 134.7, *p* < 0.001), over-reacting to situations (29.3% vs. 19.9%; *X*^2^ (3) = 99.5, *p* < 0.001), having a lot of nervous energy (32.5% vs. 22.9%; *X*^2^ (3) = 164.7, *p* < 0.001), getting agitated (24.6% vs. 20.0%; *X*^2^ (3) = 24.9, *p* < 0.001), finding it difficult to relax (34.1% vs. 23.7%; *X*^2^ (3) = 115.8, *p* < 0.001) and feeling rather touchy (29.4% vs. 18.2%; *X*^2^ (3) = 107.5, *p* < 0.001). Women also were more intolerant of anything that kept them from getting on with what they were doing (23.9% vs. 18.1%; *X*^2^ (3) = 45.0, *p* < 0.001). 

#### 5.1.3. Depression

The depression subscale of the DASS-21 revealed significant differences between women and men (*F*(1, 6863) = 22.0, *p* < 0.001). Women, on average (mean = 9.3, SD = 9.7), scored higher, indicating more depressive symptoms than men (mean = 7.9, SD = 9.6) (see Table 3). Overall, the scores indicated that men endorsed minimal depressive symptoms, while women exhibited mild depressive symptoms. A greater proportion of women could not experience positive feelings at all (14% vs. 12.5%; *X*^2^ (3) = 16.9, *p* < 0.005), could not work up the initiative to do things (26.0% vs. 21.7%; *X*^2^ (3) = 32.0, *p* < 0.001) and felt that they had nothing to look forward to (13.9% vs. 12.5%; *NS*). Women were more likely to endorse feeling down-hearted and blue (22.3% vs. 17.2%; *X*^2^ (3) = 97.2, *p* < 0.001) and feeling unenthusiastic (17.8% vs. 15.9%; *X*^2^ (3) = 20.6, *p* < 0.001). Furthermore, more women endorsed feeling not worth as much as a person (13.3% vs. 11.0%; *X*^2^ (3) = 11.5, *p* < 0.01) and that life was meaningless (10.2% vs. 9.7%; *NS*).

#### 5.1.4. Anxiety

Based on the GAD-7 responses, there was a significant gender difference (*F*(1, 6863) = 126.5, *p* < 0.001). Women, on average (mean = 6.2, SD = 5.2), scored higher, indicating more generalized anxiety than men (mean = 4.5, SD = 5.2) (see Table 4). Overall, scores indicated that men were endorsing minimal anxiety, while women exhibited mild anxiety. Women were more likely to endorse feeling nervous, anxious or on edge (19.9% vs. 14.0%; *X*^2^ (3) = 144.5, *p* < 0.001), and not being able to stop worrying more than half or nearly every day (16.7% vs. 12.0%; *X*^2^ (3) = 109.7, *p* < 0.001). A greater proportion of women also worried too much (20.7% vs. 13.9%; *X*^2^ (3) = 159.0, *p* < 0.001), had trouble relaxing (21.4% vs. 14.8%; *X*^2^ (3) = 159.2, *p* < 0.001), were too restless to sit still (14.6% vs. 11.3%; *X*^2^ (3) = 34.0, *p* < 0.001) and were easily annoyed or irritable (19.7% vs. 14.1%; *X*^2^ (3) = 120.6, *p* < 0.001). Furthermore, a greater proportion of women felt afraid, as if something awful might happen (18.0% vs. 13.1%; *X*^2^ (3) = 97.5, *p* < 0.001).

#### 5.1.5. Sleep Quality

On average, participants slept 7.1 h per 24 h period (SD = 1.5). The results on the RU-SATED scale indicate relatively good overall sleep quality (mean = 8.1, SD = 2.5) (see Table 5). Not many participants reported taking prescribed or over-the-counter medicine (16.0%) to help with sleep. However, 34.6% reported using natural sleep aids or therapies such as relaxation techniques and herbal supplements. 

There was no significant difference in overall sleep quality between women and men (*p* = 0.08). However, certain sleep quality dimensions did differ significantly, including regularity (*X*^2^ (2) = 11.4, *p* = 0.005), satisfaction (*X*^2^ (2) = 50.9, *p* < 0.001), alertness (*X*^2^ (2) = 12.3, *p* = 0.005) and duration (*X*^2^ (2) = 10.8, *p* = 0.005). Women were more likely to endorse regularity (83.5% vs. 82.3%) and alertness (85.1% vs. 81.8%), while men were more likely to endorse satisfaction (75.2% vs. 81.7%) and duration (91.0% vs. 92.8%). Additionally, women were more likely than men to endorse taking sleep medications (16.9% vs. 12.1%) and using natural sleep aids or therapies (37.9% vs. 22.1%).

### 5.2. COVID-19 Exposure and Coping during the Quarantine 

With regard to exposure to the COVID-19, few participants endorsed currently having symptoms of COVID-19 but not being tested for themselves (2.2%) or a person in their home (1.6%). A slightly larger proportion indicated that they (9.1%) or a household member (5.2%) had had symptoms but had not been tested. Less than 1% of the participants and household members had tested positive for COVID-19 at the time of the survey. Similarly, less than 1% of the participants and household members had tested positive for COVID-19 and no longer had it at the time of the survey, received medical treatment due to severe symptoms, had to stay in the hospital due to COVID-19 or had household members who died due to COVID-19. A larger proportion of participants (2.4%) endorsed having a close friend or family member die from COVID-19 or having a household member (1.7%) have a close friend or family member die from COVID-19. A series of chi-squared tests suggested that men and women mostly reported no differences in COVID-19 exposure variables, with the exception of men reporting at a slightly higher rate than women that someone in their home had been tested and currently had COVID-19 (0.5% vs. 0.2%, *p* = 0.041), that they themselves had tested positive for COVID-19 but no longer had it (0.7% vs. 0.3%, *p* = 0.040), and that they had experienced the death of a close friend or family member from COVID-19 (3.1% vs. 2.2%, *p* = 0.040).

Within the Brief COPE, significant gender differences were noted with regard to self-distraction (*X*^2^ (3) = 93.0, *p* < 0.001), active coping (*X*^2^ (3) = 40.0, *p* < 0.001), use of emotional support (*X*^2^ (3) = 212.4, *p* < 0.001), behavioral disengagement (*X*^2^ (3) = 24.3, *p* < 0.001), denial (*X*^2^ (3) = 14.5, *p* < 0.005), venting (*X*^2^ (3) = 164.5, *p* < 0.001), use of instrumental support (*X*^2^ (3) = 193.5, *p* < 0.001), positive reframing (*X*^2^ (3) = 143.9, *p* < 0.001), self-blame (*X*^2^ (3) = 74.1, *p* < 0.001), planning (*X*^2^ (3) = 52.1, *p* < 0.001), acceptance (*X*^2^ (3) = 18.8, *p* < 0.001) and religion (*X*^2^ (3) = 159.5, *p* < 0.001). There were no significant differences between women and men with regard to substance use or humor as coping mechanisms. A greater proportion of women were turning to work and other activities to keep their mind off things (55.7% vs. 44.3%). Women were more likely to cope by concentrating on doing something about the situation (55.8% vs. 49.4), getting emotional support from others (40.3% vs. 24.7%), refusing to believe that this had happened (9.7% vs. 7.8%), venting (34.7% vs. 22.9%), and getting help and advice from others (33.7% vs. 21.8%). A greater proportion of women compared with men endorsed giving up trying to deal with it a medium amount or a lot (14.0% vs. 11.1%) and trying to see it in a different light to make it seem more positive (60.6% vs. 47.6%). Further, women were more likely to criticize themselves (26.3% vs. 20.3%), try coming up with a strategy about what to do (58.1% vs. 52.0), accept the reality of COVID-19 happening (83.7% vs. 82.9%) and find comfort in religion or spiritual beliefs (37.8% vs. 23.7%).

## 6. Discussion

Our data analyses of respondents from 59 countries explored gender differences in the effects of isolation during the spread of COVID-19 on sleep, mood and coping responses. We found that compared with males, females presented with higher levels of trauma-related distress; had a harder time decompressing; had more symptoms of depression, anxiety and stress; showed decreased frustration tolerance; and reported lower quality of sleep and an increased likelihood of taking sleep medication or other natural sleep remedies. Overall, females tended to be more vulnerable during the pandemic in developing symptoms consistent with various forms of mental disorders such as depression, anxiety and PTSD.

Mental health problems are commonly more prevalent in females than males [73,74]. As an example, females are about twice as likely as males to develop depression during their lifetime [22]. Females are also more vulnerable to developing psychological symptoms (e.g., anxiety) after a stressful event or trauma [75]. In this sense, the COVID-19 pandemic entailed traumatic elements that might exacerbate the vulnerability of this population to suffer a psychiatric disorder. The reasons behind this gender gap are quite complex and might be attributed to genetics or hormonal factors [76,77], or to structural gender inequity at the societal level [78]. 

Females are also more prone to worry and rumination [79], both cognitive features strongly associated with anxiety and depression disorders. Preliminary results from the recent KFF coronavirus poll [45] reported that women worried more as primary caregivers for family health issues and financial loss due to workplace closure or reduced hours in areas of the retail, service-oriented and healthcare workforce [43,80,81]. Gender role differences also seem to play a role in the vulnerability of females to suffering mental health problems [22]. Women of childbearing age reported greater distress over reproductive health issues during the COVID-19 pandemic [44,82]. Furthermore, confinement due to lockdown conditions fostered an increase in relationship discord and even gender violence. 

It appears that theories on neurobiological responses and gender in the face of a pandemic may need to focus on defining a differential response to empathy and anxiety between men and women. Furthermore, individuals should be encouraged to implement preventive self-protective measures, which may well differ according to gender. In our study, while women were more vulnerable than men to mood symptoms during the pandemic, they also were more likely to exhibit adaptive coping responses to COVID-19 than men. Women were more likely to seek instrumental and emotional support, plan, attempt cognitive restructuring and re-framing of their problems, and use spirituality as a “grounding” strategy, which is similar to the findings of Liu and colleagues [46] in a Chinese population. Additionally, exposure cannot possibly have accounted for the gender differences we found in psychological adjustment because there were either no significant gender exposure differences in most comparisons or, when there were, men reported greater exposure. Further, the exposure base rates were extremely low and, as a result, examinations of differential exposure on gender differences in psychological adjustment by global region would be fraught with too much sample-specific error to be valid.

Designing effective mental health promotion strategies is crucial to fight against the stigma of mental health illnesses and to promote opportunities for help and early detection, especially for the female population. Easily accessible mental health services are critical during periods of prolonged quarantine, especially for those who are in urgent need of psychological support and those with limited economical and other sources of support. Since in-person health services have been limited and delayed as a result of the COVID-19 pandemic, delivery of telehealth mental health services is essential and can be delivered in the format of online services or hotlines [83]. Monetary support (e.g., beneficial funds, wages, subsidies), housing and psychiatric first aid for those vulnerable are also critical and could stave off the development of mental health disorders.

### Limitations

Certain limitations apply to this study. First, the data collection involved a cross-sectional study design and, as such, causal inferences could not be made. Additionally, all data were collected via online questionnaires independently by the study participants, which raises two concerns: (1) individual responses in self-assessment vary in objectivity when supervision from a qualified clinician/interviewer is absent, and (2) individuals with poor internet accessibility were likely not included in the study, creating a selection bias in the sample studied. Since women are, in general, more vulnerable than men to mental health problems, even prior to the stressors associated with COVID-19 social isolation periods, it is difficult to tease apart the effects of the social isolation from this predisposition and any possible interactions. Furthermore, given that it was so early into the pandemic and social distancing/shelter in place mandates, the overall exposure was very low and our analyses were not able to adequately detect gender differences in exposure. Future studies may want to include questions about mental health status prior to COVID-19 and track potential changes longitudinally in men and women.

Although the sample was generated from 59 countries, the majority of the sample was from Latin America, Europe or North America. It was difficult to obtain adequate representation from all regions in the countries participating in this study. It is possible that the results cannot be generalized to all participating countries and cannot be extended to countries that did not participate. Although a large multi-country sample can be an advantage, there are cultural differences in the expression and endorsement of symptoms of depression, anxiety and trauma-related distress. Despite this limitation in full representation across every country from which a participant took part in the current study, the sample was one of the largest and most geographically diverse collected to date on psychological adjustment during the COVID-19 pandemic. As a result, it powerfully transcends many of the small sample sizes and culturally idiosyncratic studies conducted to investigate similar topics. Future research should examine whether the effects found in this study might differ as a function of the gender role ideologies inherent in diverse global regions, as such ideologies may exacerbate or mitigate gender differences in the expression of mental health symptoms and emotions. 

Given the findings of this study, there is a clear need to develop mental health services and support for psychological difficulties within the general population, particularly for women. Further, it is essential to build these services with an eye to violence prevention and reduction of isolation. 

## 7. Conclusions

The study examined the psychological status of the general public and, more specifically, the effects of gender during the COVID-19 lockdown. A high prevalence of psychological distress was noted among women. In addition to meeting the physical health needs of the population, emphasis needs to be given to mental health support and the prevention of psychiatric disorders (e.g., major depression and PTSD, as well as anxiety), particularly in women. A combination of government policies that integrate viral risk mitigation along with prevention to alleviate risks to mental health is greatly needed. 

## Figures and Tables

**Table 1:** Demographic characteristics of the sample.

Variable	Percent
Age (mean ± SD)	42.3 (13.9)
Gender	
Women	78.8
Men	20.9
Transgender or non-binary	0.2
Marital status	
Single (never married)	35.5
Married/domestic partnership	51.1
Widowed	1.9
Divorced/separated	11.5
Employment	
Employed/engaged	80.1
Unemployed	19.9
Household	
One person	11.7
More than one person	88.3

**Table 2:** Stress symptoms in men and women in the general population during COVID-19.

Stress Symptom	Gender	Response Category (*n* = 6865)			
		Did Not Apply to Me at All *n* (%)	Applied to Some Degree *n* (%)	Applied to a Considerable Degree *n* (%)	Applied Very Much *n* (%)
I found it hard to wind down	Female	1316 (24.3)	2020 (37.2)	1413 (26.0)	676 (12.5)
	Male	554 (38.5)	499 (34.7)	288 (20.0)	99 (6.9)
I tended to over-react to situations	Female	1640 (30.2)	2192 (40.4)	1141 (21.0)	452 (8.3)
	Male	619 (43.0)	534 (37.1)	222 (15.4)	65 (4.5)
I felt that I was using a lot of nervous energy	Female	1584 (29.2)	1942 (35.8)	1245 (22.9)	654 (12.1)
	Male	659 (45.8)	451 (31.3)	252 (17.5)	78 (5.4)
I found myself getting agitated	Female	2373 (43.7)	1716 (31.6)	906 (16.7)	430 (7.9)
	Male	719 (49.9)	432 (30.0)	215 (14.9)	74 (5.1)
I found it difficult to relax	Female	1527 (28.1)	2048 (37.8)	1168 (21.5)	682 (12.6)
	Male	596 (41.4)	502 (34.9)	251 (17.4)	91 (6.3)
I was intolerant of anything that kept me from getting on with what I was doing	Female	2108 (38.9)	2022 (37.3)	906 (16.7)	389 (7.2)
	Male	693 (48.1)	487 (33.8)	187 (13.0)	73 (5.1)
I felt that I was rather touchy	Female	1700 (31.3)	2129 (39.2)	1049 (19.3)	547 (10.1)
	Male	633 (44.0)	545 (37.8)	180 (12.5)	82 (5.7)

**Table 3:** Depressive symptoms in men and women in the general population during COVID-19.

Depressive Symptom	Gender	Response Category (*n* = 6865)			
		Did Not Apply to Me at All *n* (%)	Applied to Some Degree *n* (%)	Applied a Considerable Degree *n* (%)	Applied Very Much *n* (%)
I couldn’t seem to experience any positive feeling at all	Female	2921 (53.8)	1747 (32.2)	586 (10.8)	171 (3.2)
	Male	862 (59.9)	398 (27.6)	138 (9.6)	42 (2.9)
I found it difficult to work up the initiative to do things	Female	1978 (36.5)	2033 (37.5)	956 (17.6)	458 (8.4)
	Male	640 (44.4)	488 (33.9)	216 (15.0)	96 (6.7)
I felt that I had nothing to look forward to	Female	3752 (69.2)	921 (17.0)	470 (8.7)	282 (5.2)
	Male	1033 (71.7)	228 (15.8)	112 (7.8)	67 (4.7)
I felt down-hearted and blue	Female	1962 (36.2)	2254 (41.5)	769 (14.2)	440 (8.1)
	Male	726 (50.4)	466 (32.4)	154 (10.7)	94 (6.5)
I was unable to become enthusiastic about anything	Female	2825 (52.1)	1634 (30.1)	637 (11.7)	329 (6.1)
	Male	844 (58.6)	367 (25.5)	158 (11.0)	71 (4.9)
I felt I wasn’t worth much as a person	Female	3796 (70.0)	906 (16.7)	428 (7.9)	295 (5.4)
	Male	1071 (74.4)	211 (14.7	99 (6.9)	59 (4.1)
I felt that life was meaningless	Female	4112 (75.8)	758 (14.0)	316 (5.8)	239 (4.4)
	Male	1112 (77.2)	189 (13.1)	82 (5.7)	57 (4.0)

**Table 4:** Anxiety symptoms in men and women in the general population during COVID-19.

Anxiety Symptom	Gender	Response Category (*n* = 6865)			
		Not at All *n* (%)	Several Days *n* (%)	More than Half the Days *n* (%)	Nearly Every Day *n* (%)
Feeling nervous, anxious or on edge	Female	1739 (32.1)	2524 (46.5)	591 (10.9)	571 (10.5)
	Male	702 (48.8)	536 (37.2)	115 (8.0)	87 (6.0)
Not being able to stop or control worrying	Female	2627 (48.4)	1823 (33.6)	549 (10.1)	426 (7.9)
	Male	920 (63.9)	347 (24.1)	102 (7.1)	71 (4.9)
Worrying too much about things	Female	1675 (30.9)	2626 (48.4)	621 (11.4)	503 (9.3)
	Male	696 (48.3)	544 (37.8)	126 (8.8)	74 (5.1)
Trouble relaxing	Female	1811 (33.4)	2455 (45.3)	548 (10.1)	611 (11.3)
	Male	736 (51.1)	491 (34.1)	121 (8.4)	92 (6.4)
Being so restless that it’s hard to sit still	Female	2886 (53.2)	1747 (32.2)	454 (8.4)	338 (6.2)
	Male	888 (61.7)	389 (27.0)	99 (6.9)	64 (4.4)
Becoming easily annoyed or irritable	Female	1832 (33.8)	2527 (46.6)	617 (11.4)	449 (8.3)
	Male	711 (49.4)	525 (36.5)	127 (8.8)	77 (5.3)
Feeling afraid, as if something awful might happen	Female	2389 (44.0)	2060 (38.0)	492 (9.1)	484 (8.9)
	Male	841 (58.4)	410 (28.5)	110 (7.6)	79 (5.5)

**Table 5:** Sleep quality in the general population during COVID-19.

Sleep Quality Dimension	Response Category (*n* = 6865)		
	Never *n* (%)	Sometimes *n* (%)	Always *n* (%)
Regularity	1149 (16.7)	2282 (33.2)	3434 (50.0)
Satisfaction	1611 (23.5)	3032 (44.2)	2222 (32.4)
Alertness	1071 (15.6)	2073 (30.2)	3721 (54.2)
Timing	524 (7.6)	1418 (20.7)	4923 (71.7)
Efficiency	1897 (27.6)	2555 (37.2)	2413 (35.1)
Duration	588 (8.6)	2044 (29.8)	4233 (61.7)

## Data Availability

The datasets used and/or analyzed during the current study available from the corresponding author on reasonable request.

## Abbreviations

ACRM	American Congress of Rehabilitation
ANOVA	Analysis of Variance
COPE	Coping Orientation to Problems Experienced
COVID-19	Coronavirus Disease 2019
CRIES-8	Children’s Revised Impact of Events Scale-8
DASS-21	Depression, Anxiety and Stress Scale-21
EPII	Epidemic–Pandemic Impacts Inventory
GAD-7	Generalized Anxiety Disorder-7
MERS	Middle East Respiratory Syndrome
NS	Not Significant
PTSD	Post-Traumatic Stress Disorder
RU-SATED	Regulatory Satisfaction Alertness Timing Efficiency Duration
SARS	Severe Acute Respiratory Syndrome
SD	Standard Deviation
WHO	World Health Organization
